# PRODUCTIVE AND ACTIVE RURAL AGING: TOWARD CRITICAL PERSPECTIVES

**DOI:** 10.1093/geroni/igz038.061

**Published:** 2019-11-08

**Authors:** Mark Skinner, Rachel Winterton, Kieran Walsh

**Affiliations:** 1 Trent University, Peterborough, Ontario, Canada; 2 La Trobe University, Bendigo, Victoria, Australia; 3 NUI Galway, Galway, Ireland, Ireland

## Abstract

Despite global trends in rural population ageing, relatively little attention within research and policy has been directed to understanding what it means for rural people, communities and institutions to be at the forefront of twenty-first century demographic change. To build understanding of rural ageing, this symposium draws together papers from four countries to provide insights in the gaps in rural ageing research – specifically the in context of productive and active rural ageing by examining rural work, retirement and volunteering through the critical perspectives of citizenship, contestation and complexity. Winterton and Warburton will explore how active citizenship trends among rural older adults support or hinder the capacity of rural settings to support health ageing. Colibaba and Skinner will discuss the contestation of rural ageing by examining a volunteer-based rural library and the emergent ‘contested spaces of older voluntarism’ whereby older volunteer negotiate their rights and responsibilities associated with ageing and volunteering in rural communities. Duvvury and Ni Leime will examine the interactions between the twin phenomena of feminisation of agriculture and the feminisation of ageing in the consequent implications for rural women’s work and retirement. Skinner and Joseph offer a critical perspective on voluntarism in ageing rural communities by examining volunteer leadership biographies as another means of understanding the contribution of older rural adults.

